# Supplementary material for: Willingness to use mental health counseling in diverse groups of Asian Americans
Source: PLoS One. 2024 Jul 26;19(7):e0306064. doi: 10.1371/journal.pone.0306064 (PMC11280139; doi:10.1371/journal.pone.0306064)
Supplement: Attachment — Submitted filename: Feedback on Journal Paper.pdf [file pone.0306064.s001.pdf]

May 3,2024

Nor Mazlina Ghazali, Ph.D
Editor, PLOS One

Enclosed is our revision of the manuscript entitled "Willingness to Use Mental Health Counseling in Diverse Groups of Asian Americans." We sincerely appreciate you, as the Academic Editor, and the Reviewers for the thoughtful comments on our work. This letter addresses how we responded to each of the concerns raised.

Comments:

1. In abstract the researcher is encouraged to insert the implication of study and suggestion to future researcher.

Comments:

2. In Introduction the researcher is encouraged to analyse the information provided in the introduction. It is not only using the information from literature review/previous study.

Comments:

3. Research Methodology
    - Researcher is encouraged to insert the term "Quantitative research design.
    - Researcher need to provide the type of sampling technique use in this study.
    - Researcher is also need to determine the number of sample size.
    - Be specific when you describe the test of data analysis. For instance: descriptive analysis (i.e: percentage, frequency, mean etc) and inferential analysis (if related)
    - Simplify your table of descriptive analysis
    - State AAQoL as instrument in measuring and please state clearly this survey used to measure what variable?

Comment:

4. Discussion on result is good, reliable and acceptable. Simplify your table of descriptive analysis.

Comment:

5. Discussion is good and align with result.

Comment:

6. Conclusion is good.
